# The Treatment of Acute Cutaneous Leishmaniasis With Oral Zinc Bisglycinate and Oral Hydroxychloroquine Sulfate

**DOI:** 10.7759/cureus.84618

**Published:** 2025-05-22

**Authors:** Adil A Noaimi, Maryam Mohamed

**Affiliations:** 1 Department of Dermatology and Venereology, College of Medicine, University of Baghdad, Baghdad, IRQ; 2 Department of Dermatology and Venereology, Baghdad Center of Dermatology and Venereology, Medical City, Baghdad, IRQ

**Keywords:** cutaneous leishmaniasis, leishman-donovan body, oral hydroxychloroquine sulfate, oral zinc bis-glycinate, sharquieˋs modified leishmania score

## Abstract

Background and objective

Cutaneous leishmaniasis (CL) is one of the most significant and endemic parasitic infections in many regions around the world, and it is caused by several species of the Leishmania parasite. It is a morbid condition that leads to stigmatization and disfiguring scars. Numerous methods have been tried to treat leishmaniasis with variable cure rates and side effects. This study aimed to compare the effects of oral zinc bisglycinate (ZnGly) and oral hydroxychloroquine sulfate (HCQ), alone and in combination, in treating acute CL.

Materials and methods

This comparative therapeutic study was conducted at the Center of Dermatology and Venereology, Medical City, Baghdad, Iraq, during the period from December 2021 to February 2023. All participants had been diagnosed through history and clinical examination, which involved skin smear in all patients and skin biopsy for some suspicious cases. Patients were classified into three groups based on the type of treatment: Group A was treated with oral ZnGly capsules 50 mg twice daily for eight weeks; Group B was administered oral HCQ tablets 200 mg twice daily for eight weeks; and Group C received a combination of both drugs for eight weeks. All patients were examined every four weeks for eight weeks, followed by every month for two months after stopping therapy. The treatment's impact on the lesions was assessed using Sharquieˋs modified Leishmania score.

Results

A total of 40 patients with acute CL were included in this study, which spanned eight weeks. The total number of lesions was 144, and their duration ranged from five to 12 weeks. Group A included 14 patients, and the cure rate was 78.6% (11); Group B included 13 patients, and the cure rate was 84.7 % (11), while Group C included 13 patients, and the cure rate was 92.3% (12). We observed a statistically significant difference in the cure rate in Group C when compared to Group A and Group B at the end of the study (p=0.034).

Conclusions

While both oral ZnGly and HCQ as monotherapy were effective in the treatment of acute CL, the combination regimen of both drugs led to higher effectiveness without any increase in the frequency of adverse effects.

## Introduction

Leishmaniasis is one of the most notable parasitic disorders and a zoonotic disease, and it is characterized by parasitized mononuclear phagocytic system [1]. Cutaneous leishmaniasis (CL) is the most prevalent clinical leishmanial manifestation [2]. It is a morbid condition that results in stigmatization and causes disfiguring scars [3]. The exact incidence of CL is not known, but it is estimated that more than one million new cases occur each year in approximately 90 countries worldwide [4]. Moreover, it is becoming a universal problem due to the surge in immigration and international travel [5]. The psychological impact of the disease is also substantial [5,6]. Its treatment primarily focuses on shortening the duration of the scars and improving the cosmetic results of its scar. Of note, patients with significant immunosuppression require special attention.

Treatment of the condition can be classified into local, which involves heat therapy, cryotherapy, pentavalent antimony (SbV) compounds, 2% zinc sulfate solution, chloroquine (CQ), etc. [5]; and systemic treatment, which employs zinc sulfate [5], CQ [7-9], pentavalent antimony, etc. [8,10]. Zinc bisglycinate (ZnGly) is a zinc amino acid chelate (organic form), comprising one zinc molecule bound to two (bis = two) molecules of the amino acid glycine. It is a white crystalline powder with a molecular weight of 233.5 g/mole (molecular formula: C4H12N2O5Zn, exact mass: 232.003763 g/mol) [11]. Hydroxychloroquine sulfate (HCQ) (brand name Plaquenil) is a 4-aminoquinoline antimalarial agent, which has recently emerged as an advantageous therapy in a wide range of diseases other than malaria [12]. Apart from its antimalarial activity, HCQ has anti-infectious, anti-proliferative, anti-inflammatory/immunomodulatory, and photo-protective effects [13]. In this study, we aimed to compare the effectiveness and safety profile of oral ZnGly and oral HCQ in the treatment of acute cutaneous leishmaniasis, alone and in combination with each other.

## Materials and methods

Study design and setup

This was an analytic, interventional, prospective cohort comparative study conducted at the Center of Dermatology and Venereology, Medical City, Baghdad, Iraq, during the period from December 2021 to February 2023. Detailed history was taken from patients, including age, gender, residential status, occupation, duration of lesions, history of previous treatments, and past medical history. Cutaneous examination was performed for site, size, and type of the lesions. All patients had been diagnosed with acute CL through history and clinical examination, which involved skin smear for all patients and skin biopsy for some suspicious cases. Nature of the disease, its complications and prognosis, type and duration of treatment, and its associated adverse effects were explained in detail to each patient before starting therapy, and formal consent was taken from each patient regarding therapy and photography.

Ethical approval

Ethical approval for this study was obtained from the Scientific Committee of the Scientific Council of Dermatology and Venereology of the Iraqi Board for Medical Specializations.

Inclusion and exclusion criteria

Inclusion Criteria

The inclusion criteria were as follows: patients with more than four lesions of substantial size or individual lesion(s) measuring ≥5 cm; patients with sporotrichoid lesions or lesions close to eyes or above joints; patients aged 18 years and above.

Exclusion Criteria

The exclusion criteria were as follows: patients who had received anti-leishmaniasis treatment locally or systemically in the last four weeks before this study; patients on prolonged corticosteroid therapy or other immunosuppressive therapies; patients with chronic ailments like diabetes mellitus, liver disease, renal disease and other chronic, and immunosuppressive illnesses; patients with cardiac diseases and ophthalmological problems; pregnant patients, lactating women, children, and patients below 18 years of age; and patients who did not fully comply with treatment.

Data collection and analysis

The patients were categorized into three groups according to the therapeutic regimen. Group A: patients were treated with oral ZnGly capsule (Mada-Zinc Forte) 50 mg twice daily after meals for eight weeks; it was purchased from Madamar Company, Warsaw, Poland. Group B: patients were treated with oral HCQ sulfate tablet (Dolquine R) 200 mg twice daily for eight weeks (manufactured by Laboratorios Rubió, Castellbisbal, Spain. Group C: patients were treated with a combination of both drugs for eight weeks. Laboratory tests, including liver and renal function tests, viral serology, and complete blood count, were performed at baseline (before starting therapy) and at the end of the study. For patients who received HCQ (either alone or in combination with ZnGly), slit lamp and fundoscopic examination were done for the assessment of visual acuity and visual field testing.

The erythema, diameter, crustation, and ulceration of each lesion in the three groups were evaluated. The diameter of each lesion was measured by noting the induration and using a Vernier caliper to measure its diameter in centimeters. The response to treatment was assessed using Sharquie's modified leishmania score. Patients in all three groups had reassessment every four weeks for a total treatment period of eight weeks. During each visit, the parameters of the lesions were re-evaluated to determine the score, assess the response to the therapy, and record any local or systemic adverse effects. Subsequently, patients were monitored monthly for an additional two months after discontinuing therapy, as a follow-up to record any recurrence of lesions. Photographs of each lesion were taken at different time intervals, with fixed illumination, using an iPhone 11 (Apple Inc., Cupertino, CA) camera.

## Results

A total of 40 patients were included in this study (14 in Group A, 13 in Group B, and 13 in Group C). The age of the studied sample ranged from 18 to 63 years. The cohort had a male predominance: 29 (72.5%) patients were males and 11 (27.5%) were females (male-to-female ratio of 2.6:1). Among 40 patients, 18 (45%) were soldiers. As for residential status, 24 (60%) were from rural areas, whereas 16 (40%) hailed from urban areas; 15 (37.5%) had a family history of CL. There was no previous history of similar lesions in any one of the included patients. Among 40 patients, 12 (30%) had received treatment in more than four weeks before starting the study. The duration of lesions was between 5 and 12 weeks. There was no statistically significant difference among the three groups regarding age, sex, and duration of lesions (Table 1).

**Table 1:** Sociodemographic features of the study participants ^*^Statistical significance was set at a p-value ≤0.05 SD: standard deviation

Variable		Group A (n=14)	Group B (n=13)	Group C (n=13)	P-value^*^
Age, years, mean ±SD		38.0 ±5.8	36.0 ±6.1	39.0 ±6.0	0.164
Sex, n (%)	Female	4 (28.6%)	3 (23.1%)	4 (30.8%)	0.902
	Male	10 (71.4%)	10 (76.9%)	9 (69.2%)	
Duration, weeks, mean ±SD		6 ±1.1	8 ±2.1	7 ±1.5	0.237

Among a total number of 144 lesions, with a size ranging between 5 and 8 cm, 121 (84%) lesions were of dry type, nine (6.3%) wet, eight (5.5%) satellite, and six (4.2%) sporotrichoid. As for the distribution of lesions according to site, 83 (57.6%) lesions were located in the upper limbs, 47 (32.6%) in the lower limbs, 11 (7.6%) in the trunk, and three (2%) in the face. No statistically significant difference was detected among the three study groups regarding lesion type and site (Table 2).

**Table 2:** Distribution of lesions in the study groups according to type and site ^*^Statistical significance was set at a p-value ≤0.05

Variable		Group A (n=55), n (%)	Group B (n=45), n (%)	Group C (n=44), n (%)	P-value^*^
Lesion type	Wet	5 (9.1%)	3 (6.7%)	1 (2.3%)	0.251
	Dry	45 (81.8%)	38 (84.4%)	38 (86.4%)	
	Sporotrichoid		3 (6.7%)	3 (6.8%)	
	Satellite	5 (9.1%)	1 (2.2%)	2 (4.6%)	
Site	Face	1 (1.8%)		2 (4.6%)	0.252
	Trunk	6 (10.9%)	5 (11.1%)		
	Upper limb	27 (49.1%)	30 (66.7%)	26 (59.1%)	
	Lower limb	21 (38.2%)	10 (4.5%)	16 (36.4%)	

**Figure 1:**
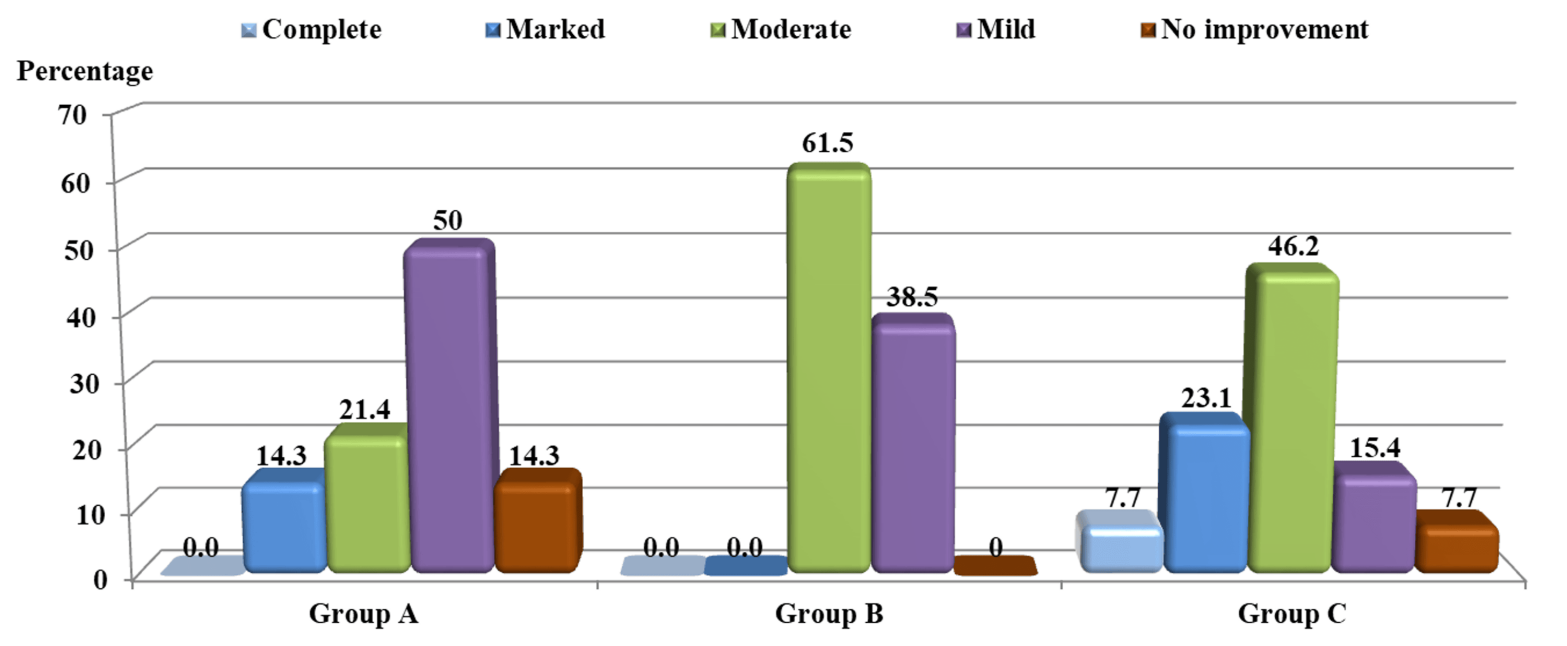
Level of response to treatment in the study groups after four weeks Group A: zinc bisglycinate therapy; Group B: hydroxychloroquine sulfate therapy; Group C: combined therapy

**Figure 2:**
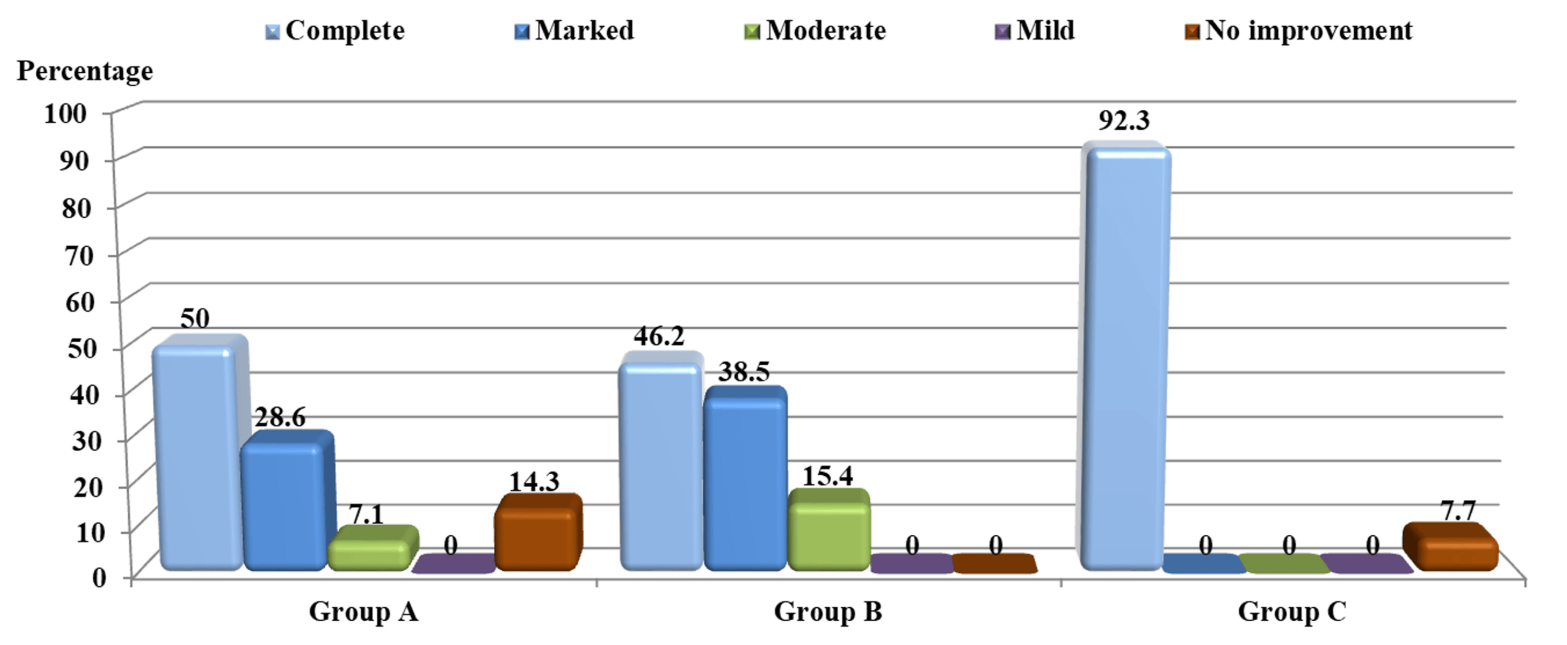
Level of response to treatment in the study groups after eight weeks Group A: zinc bisglycinate therapy; Group B: hydroxychloroquine sulfate therapy; Group C: combined therapy

**Figure 3:**
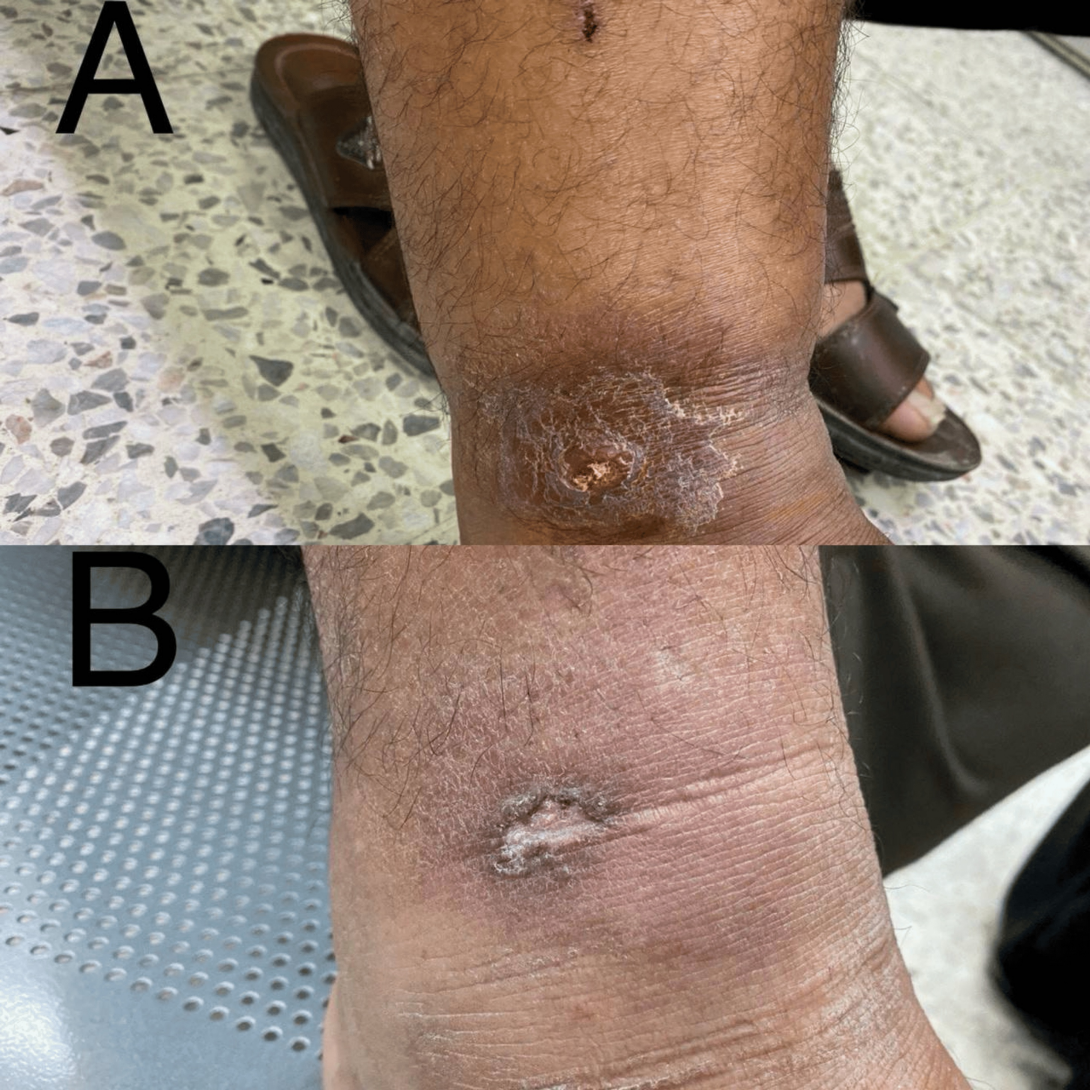
A 63-year-old male patient with cutaneous leishmaniasis in the right ankle before therapy (A) and after eight weeks of therapy with oral zinc bisglycinate

The response to each treatment was as follows: In Group A (ZnGly), after four weeks of starting therapy, the mean score was 12.6 ±3.0, and it was statistically significantly different in comparison to before treatment (p<0.001). After eight weeks of treatment, the mean score was 6.8 ±4.6, which was statistically significantly different in comparison to before treatment (p<0.001). Regarding the treatment response in each visit during treatment, after four weeks of therapy, two (14.3%) patients showed no improvement, seven (50.0%) showed mild improvement, three (21.4%) moderate improvement, and two (14.3%) marked improvement (Figure 1). And after eight weeks, two (14.3%) patients showed no improvement, one (7.1%) moderate improvement, four (28.6%) marked improvement, and seven (50.0%) complete improvement (Figure 2). Hence, 11 (78.6%) patients experienced a cure (Figures 2, 3), and no significant side effects, apart from fatigue, loss of appetite, and nausea.

**Figure 4:**
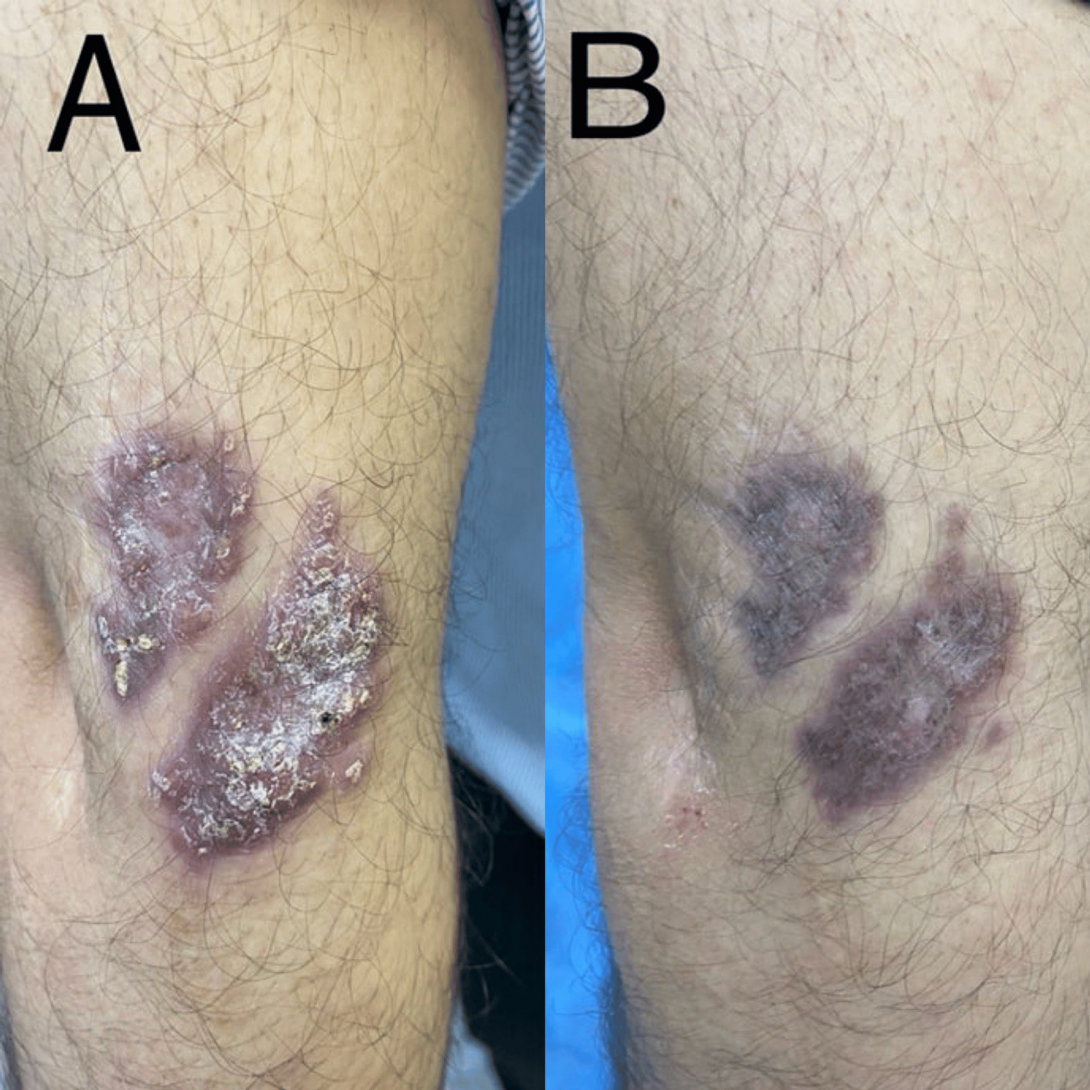
A 44-year-old male patient with cutaneous leishmaniasis in the right arm before therapy (A) and after eight weeks of therapy with hydroxychloroquine sulfate (B)

In Group B (HCQ), after four weeks of starting therapy, the mean score was 11.4 ±2.0; it was statistically significantly different in comparison to before treatment (p<0.001). After eight weeks of therapy, the mean score was 5.7 ±2.4, which was statistically significantly different in comparison to before treatment (p<0.001). Regarding treatment response in each visit during treatment, after four weeks of therapy, five (38.5%) patients showed mild improvement and eight (61.5%) moderate improvement (Figure 1). And after eight weeks, two (15.4%) patients showed moderate improvement, five (38.5%) marked improvement, and six (46.2%) complete improvement (Figure 2). Hence, 11 (84.7%) patients experienced a cure (Figures 2, 4), with no remarkable side effects, apart from nausea and dizziness.

**Figure 5:**
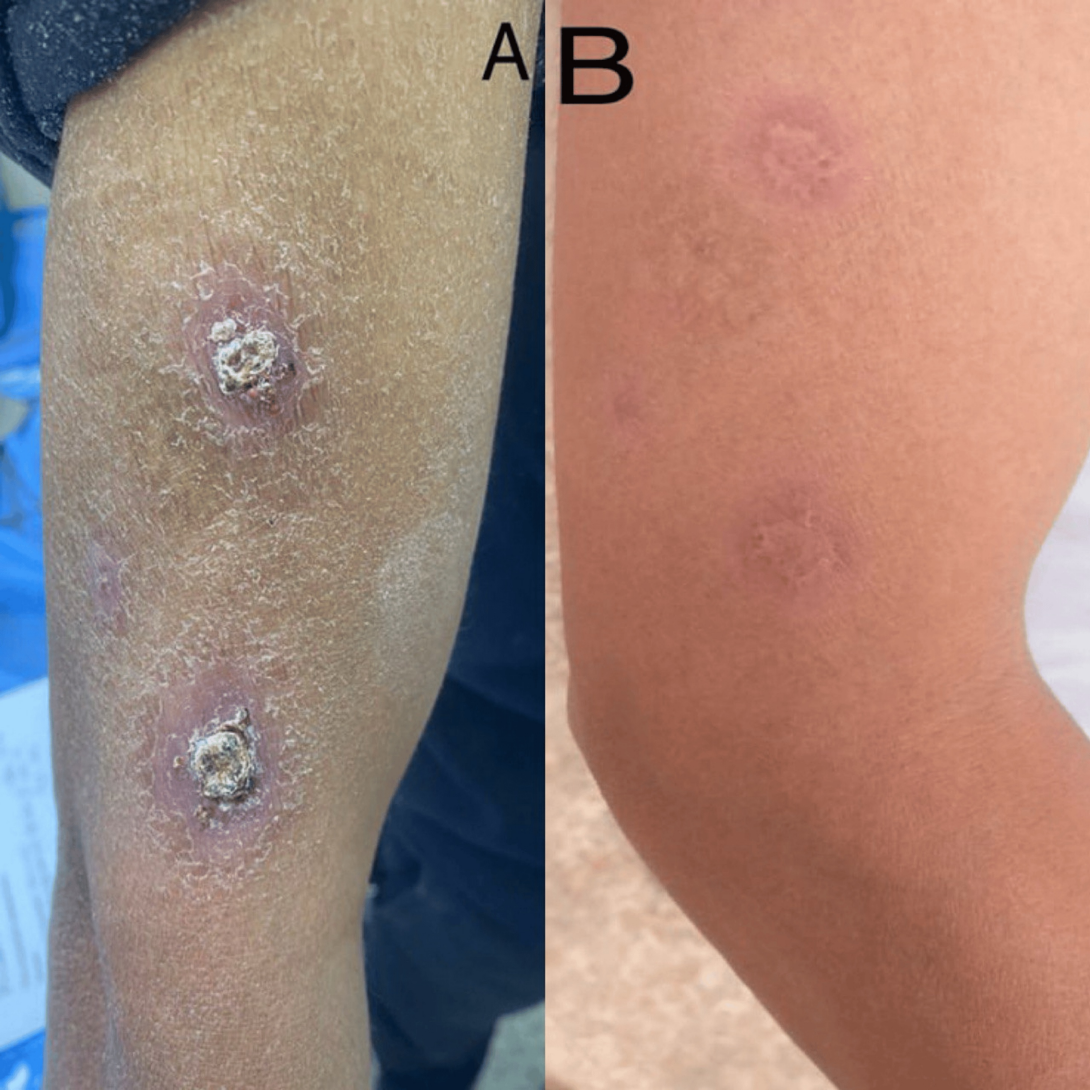
A 50-year-old male patient with cutaneous leishmaniasis in the right arm before therapy (A) and after eight weeks of combined therapy (B)

In Group C (combination), after four weeks of initiation of therapy, the mean score was 9.7 ±3.6; it was statistically significantly different in comparison to before treatment (p<0.001). After eight weeks, the mean score was 3.7 ±3.8, which was statistically significantly different in comparison to before treatment (p<0.001). As for treatment response in each visit during therapy, after four weeks, one (7.7%) patient showed no improvement, two (15.4%) mild improvement, six (46.2%) moderate improvement, three (23.1%) marked improvement, and one (7.7%) complete improvement (Figure 1). And after eight weeks, one (7.7%) patient showed no improvement, while 12 (92.3%) showed complete improvement (Figure 2). Hence, 12 (92.3%) patients experienced a cure (Figures 2, 5), with no significant side effects, apart from headache.

When comparing Group A and Group C regarding the mean score, there was a statistically significant difference after four and eight weeks of starting treatment (p=0.041, p=0.017, respectively (Table 3). When we compared Group B and Group C based on the mean score, there was no statistically significant difference after four weeks of therapy (p=0.205), but there was a statistically significant difference at eight weeks (p=0.008) (Table 3). As for the comparison between Group A and Group B concerning mean score, there was no statistically significant difference after four and eight weeks of treatment (p=0.171, p=0.844, respectively) (Table 3). As for the cure rate, it was higher in Group C than both Group A and Group B, with statistically significant differences at four and eight weeks of starting therapy (p=0.025, p=0.034, respectively). No statistically significant difference was detected among the three study groups regarding side effects (p=0.782).

**Table 3:** Comparison of p-values in terms of mean total scores among the three groups in each visit ^*^Statistical significance was set at a p-value ≤0.05

Visit		Group A	Group B	Group C
Score after four weeks, mean ±SD		12.6 ±3.0	11.4 ±2.0	9.7 ±3.6
P-value	Group A vs. Group B	0.171		
	Group A vs. Group C	0.041^*^		
	Group B vs. Group C	0.205		
Score after eight weeks, mean ±SD		6.8 ±4.6	5.7 ±2.4	3.7 ±3.8
P-value	Group A vs. Group B	0.844		
	Group A vs. Group C	0.017^*^		
	Group B vs. Group C	0.008^*^		

## Discussion

Various therapies have been tried in the treatment of CL, both local and systemic [5]. There is a pressing need to identify oral, safe, available, affordable, and effective treatment options for CL [14]. Several studies have shown variable response rates in the treatment of CL by different systemic drugs, which could be attributed to the probability of spontaneous healing of lesions and the different parameters that were used to assess the cure of lesions. The last decade witnessed the emergence of new species with resistance to the available modalities of treatment, both locally and systemically. This study was carried out to compare the effectiveness of oral ZnGly and oral HCQ sulfate in the treatment of acute CL, alone and in combination. This is the first Iraqi study to analyze the use of ZnGly and HCQ alone and in combination in the treatment of CL.

We observed a cure rate of 78.6% with oral ZnGly treatment in our cohort, with no significant side effects. In several clinical trials previously conducted in Iraq by Sharquie et al. to assess the effectiveness of oral zinc sulfate in the treatment of of CL, in doses of 2.5, 5, and 10 mg/kg/day for 45 days, the observed cure rates were 83.9%, 93.1%, and 96.9%, respectively [5]. The variance in cure rate in our study compared to these previous studies might be due to the use of Sharquie's modified Leishmania score (used in the present study), as it is more impartial than the semi-topical old Sharquie's Leishmania score (used in the previous studies) [5]. Another study from Iraq in 2015 by Sharquie et al. used oral zinc sulfate 10mg/kg/day for six weeks; Sharquie's modified Leishmania score was used to evaluate the results after starting therapy, and the cure rate was 60% with no significant side effects [5]. Hence, ZnGly is higher in terms of absorption and more bioavailable than zinc sulfate [15,16], with a higher cure rate in the treatment of CL as demonstrated in the present study.

In our study, the cure rate with oral HCQ therapy was 84.7%, with no appreciable adverse effects. The use of oral CQ in CL has shown a cure rate of 100% with no significant adverse effects in multiple studies conducted in Pakistan in 2007 by Khan et al. (oral CQ 250 mg TDS for 20 days), in 2014 by Hanif et al. (oral CQ 250 mg once daily; the maximum duration of treatment was not more than four months), and in 2017 by Hanif et al. (oral CQ 250 mg BID for three months) [7-9]. However, in another study conducted in Pakistan in 2018 by Farooq et al. on the effectiveness of oral CQ with a dose of 250 mg BID for 28 days in CL, the cure rate was 56% with no significant adverse effects [8]. Hence, CQ is dose and duration-dependent. Although it is effective in the treatment of CL, HCQ is better than CQ in terms of the safety profile, not only in the general population but also among special cases, including pregnant women and those who suffer from renal failure [12].

The use of SbV against CL has been associated with a cure rate of 65% in a study conducted in Pakistan in 2007 by Saleem et al. and (84%) in a study conducted in Pakistan in 2018 by Farooq et al., with fewer side effects [8,10]. Although it is effective in the treatment of CL, its use is limited due to the need for parenteral administration with daily injections for 20-30 days and toxic effects in several organs. Moreover, it cannot be used in pregnant females; healing with Sbv treatment takes longer, and increasing resistance to Sbv therapy is a serious concern in CL control [17]. When compared to oral allopurinol tablets for a two-week duration, the cure rate was 70% with no significant side effects, in a study conducted in Pakistan in 2001 by Mashhood et al. [8]. It is comparable to the cure rate in the present study, and its use can lead to a faster cure than HCQ.

Comparison with antifungal drugs

Multiple studies have been conducted involving the use of antifungal drugs against CL, with fewer side effects.

Oral Itraconazole

In a study conducted in Pakistan in 2007 by Saleem et al. on the effectiveness of itraconazole (100 mg BID for six to eight weeks), the cure rate was 75% with fewer side effects [10]. It aligns with the cure rate of the present study with the same duration of treatment. But sporotrichoid leishmaniasis did not respond to itraconazole [10], in contrast to HCQ, which worked on sporotrichoid lesions in the present work.

Oral Ketoconazole

In a study conducted in Iraq in 2015 by Sharquie et al., the cure rate was 50% with no appreciable side effects [5]

Oral Fluconazole

With the use of oral fluconazole capsule 200 mg daily for six weeks for Leishmania major CL, the cure rates were 59% and 48.3% with no significant adverse effects in studies conducted in Saudi Arabia in 2002 by Alrajhi et al. and in Iran in 2011 by Emad et al., respectively [5,18]. And with the use of oral fluconazole capsule 400 mg daily for six weeks in Leishmania major CL, the cure rate was 81% in the study by Emad et al. However, its use was associated with the rise of serum creatinine, elevation of liver enzymes, cheilitis, and nausea [18]. Hence, oral fluconazole of a 200 mg daily dose is associated with variable response in CL, and at higher doses, it is associated with significant adverse effects that limit its use.

Rifampicin use (10 mg/kg/day) in CL for four weeks has been associated with a cure rate of 93.8% with very low side effects, in a study conducted in Saudi Arabia in 2019 by AlSohaimi et al. [19]. It is similar to the cure rate in the present work, and it can provide an earlier cure than HCQ. As for dapsone (100-200 mg daily), the cure rates were (80%, 48.8%, and 66% in studies conducted in India in 1991 by Dogra et al. for six weeks, in Kuwait in 2007 by Al-Mutairi et al. for six to eight weeks, and in Pakistan in 2018 by Ahmed et al. for a maximum of 80 days, respectively. Moreover, it was well tolerated, cost-effective, and easily available [20,21]. However, its response rates varied.

Regarding the use of doxycycline 200 mg daily, the cure rates were 71% and 92% in studies conducted in Tunisia in 2007 by Masmoudi et al. for 15-30 days and in Pakistan in 2017 by Hanif et al. for three months, respectively. Phototoxicity is considered to be the main side effect associated with doxycycline, but it was not reported in the mentioned studies [7,22]. The cure rate in these studies aligns with our rate, and the drug can provide an earlier cure than HCQ. In the present study, the cure rate with combined therapy was 92.3%, with no notable side effects. In a study conducted in Iraq in 2015 by Sharquie et al., which used a combination of oral zinc sulfate 10 mg/kg/day and ketoconazole tablet 200 mg BID daily for six weeks, the cure rate was 96%, with no notable side effects [5], which is similar to the cure rate in the present study.

Limitations

This study has a few limitations, primarily its small sample size, which could be attributed to the decrease in the cases of CL due to climate-related variables [lack of rain and floods, which affect the life cycle of rodents (animal reservoir) and sand fly vector]. Also, several patients insist on receiving intralesional injection of Pentostam as it is the most popular method among people to treat CL.

## Conclusions

Based on our findings, a combination regimen comprising oral ZnGly and HCQ can be an effective, safe, and cost-effective novel option in the treatment of CL, with no notable adverse effects. Although treatment with ZnGly or HCQ as monotherapy can also achieve high cure rates, combined therapy demonstrated better patient outcomes.
